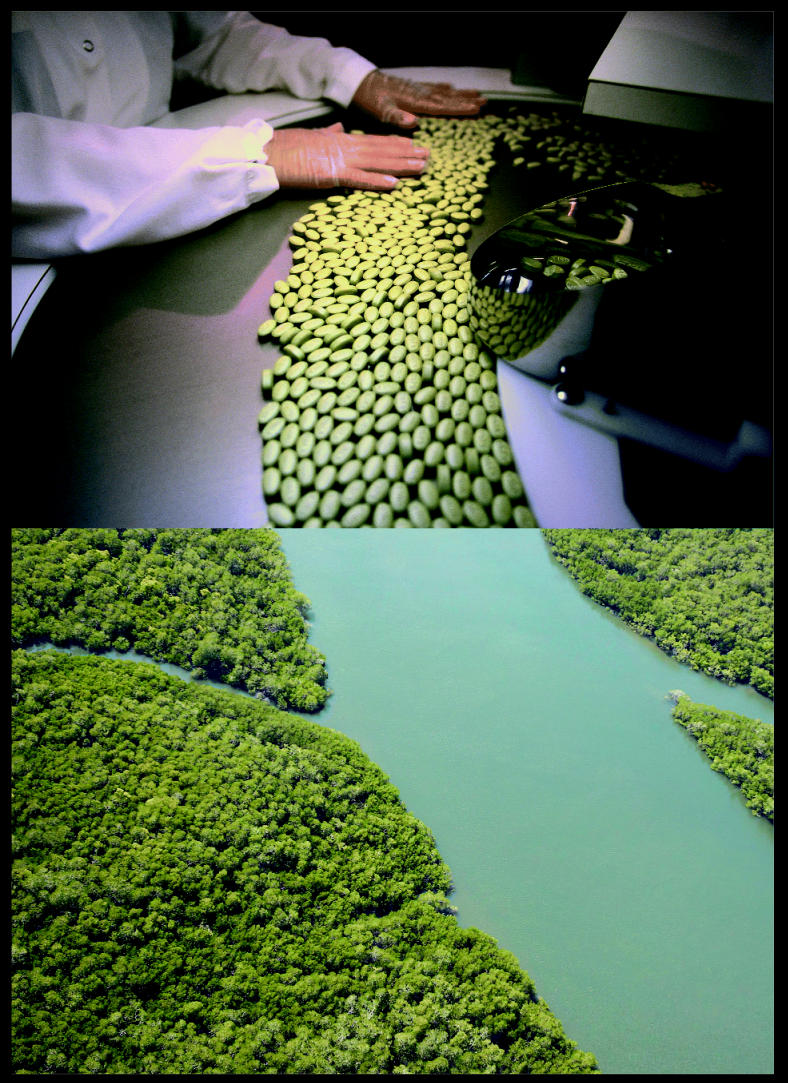# **Damming the Flow** of Drugs into Drinking Water

**DOI:** 10.1289/ehp.113-a678

**Published:** 2005-10

**Authors:** Pat Hemminger

Roughly 100 pharmaceuticals have now been identified in rivers, lakes, and coastal waters through out Europe and the United States in concentrations of parts per billion to parts per trillion. The first major European studies on this topic—in journals such as volume 67, issue 1–4 (1997) of the *International Journal of Environmental Analytical Chemistry* and the November 1998 issue of *Water Research*—examined German ground and surfaces waters, and found occurrences of drugs including cholesterol regulators, analgesics, and antiseizure medications. Since that time, numerous other studies have documented the presence of pharmaceuticals, including potential endocrine disruptors, in other locales as well.

So far there is no evidence of adverse human health effects due to traces of pharmaceuticals in water. But scientists have linked certain pharmaceuticals with disturbing ecosystem changes. For example, in volume 8 (1994) of *Chemistry and Ecology*, researchers demonstrated that the feminization of fish—male carp and trout producing vitellogenin, an egg protein usually found only in females—was associated with exposure to sewage effluent now known to contain ethinyl estradiol, the active ingredient in birth control pills.

There is much concern about what is not known: ecotoxicity data are available for less that 1% of human pharmaceuticals, according to estimates published in the April 2004 issue of *Regulatory Toxicology and Pharmacology*. Today, intensive research is under way to investigate the effect of human medications on the environment.

In 1999, in response to these concerns, the European Medicines Agency (EMEA) began drafting guidance that outlined an environmental risk assessment procedure to accompany pharmaceutical companies’ applications to market new drugs in Europe. The latest draft was published in January 2005, after several revisions, and the public comment period closed in April 2005. Scientists and pharmaceutical companies alike hope the guidance will be finalized later this year.

The proposed European guidance is the first to recommend long-term ecotoxicity testing for environmental risk assessment of pharmaceuticals from the outset of the proposed testing program (in contrast, U.S. Food and Drug Administration [FDA] requirements for chronic ecotoxicity testing come later in that agency’s assessment). The European guidance is also the first to take into account the possibility of environmental effects from extremely low concentrations of bioactive substances, such as endocrine disruptors.

If finalized, the guidance could call for substantially more testing of new drugs than has been demanded thus far. Its implementation would also generate much-needed chronic ecotoxicity data. “The main advance in this draft guideline is that we really address this issue and get more information on the toxicity of these compounds,” says Thomas Heberer, an environmental chemist at the Technical University of Berlin and coauthor of many papers on the topic, including the 1997 *International Journal of Environmental Analytical Chemistry* report.

## What the Draft Guidance Covers

The draft guidance outlines the risk assessment procedure for new active pharmaceutical substances, their metabolites, and possibly excipients (the inert substances in which a drug is delivered) if they are deemed similar to chemicals with known adverse environmental effects. It does not apply to drugs already on the market. If an environmental risk is found, the guidance recommends that the manufacturer take appropriate precautionary and safety measures to limit the product’s environmental impact. The guidance specifically recommends the labeling of pharmaceuticals when there is a possibility of an environmental risk, to educate people about how best to dispose of expired or unused medicines.

The guidance applies only to potential environmental risks that are a consequence of people storing, taking, and excreting medicines. The potential risks posed by the manufacture of drugs are not addressed, nor does the guidance apply to “orphan” drugs used only to treat rare diseases. Separate guidance governs medicinal products containing genetically modified organisms.

## Proposed EMEA Protocols

The EMEA risk assessment protocol is a tiered process that begins with a rough calculation of the aquatic predicted environmental concentration (PEC) of the new drug. During this Phase I prescreening, substances whose PEC is deemed too low to be of concern to environmental health are ruled out for further assessment. Vitamins, electrolytes, amino acids, peptides, and proteins are exempted by the guidance because they are not tailored active ingredients (unlike, for example, a drug that interacts with a receptor) and thus are deemed “unlikely to result in significant exposure of the environment.” However, the guidance does note that certain substances that are likely to cause effects at very low concentrations, such as endocrine disruptors, may need to be addressed regardless of the quantity released into the environment.

Phase II begins with Tier A testing, which aims to determine the aquatic fate and effects of the drug. Its degradability, potential to bioaccumulate, adsorption on sewage sludge, and toxicity to sewage microbial populations are evaluated from the results of standard tests also used in the FDA risk assessment. Also included in Tier A of the EMEA protocol is the long-term testing of fish, *Daphnia* (water fleas), and algae to assess the predicted “no effect” concentration (PNEC) of the new drug for each of these species. The PEC is further refined at this stage in the EMEA assessment by taking into account the pharmaceutical company’s projected sales forecast for the drug.

The risk assessment is terminated if the outcome of Tier A testing results in a PEC lower than the PNEC. However, if the PEC is greater than the PNEC in either water, sediment, the sewage treatment plant, or soil (where sewage sludge has been spread as a fertilizer), this indicates a potential risk, and further Tier B testing is initiated. These tests follow the protocol in the *European Technical Guidance Document* to further investigate the risk posed by the drug to the environment. For instance, where there is a potential risk to soil, tests would be conducted to determine the drug’s biodegradation in soil, its toxicity to soil invertebrates, and its acute effects on plants and soil microorganisms.

At this stage, data on the drug metabolism and excretion profile may be consulted to allow a more accurate calculation of the PEC and determine whether metabolites need to be tested. The EMEA guidance recommends that metabolites exceeding 10% of the drug residue should be assessed for environmental risk. If this round of testing indicates that the PEC of the drug will be greater than the PNEC, then pharmaceutical companies following the European approach must propose recommendations to limit the drug’s impact on the environment.

There are two major differences between the proposed EMEA approach and the existing FDA approach. First, the FDA protocol turns to chronic testing only if acute testing indicates a risk or if there is an indication that the drug could bioaccumulate. The latest scientific research suggests that acute testing is not a reliable indicator of all chronic effects, however, and the EMEA document reflects this finding.

Second, the trigger concentrations of pharmaceuticals that prompt risk assessment under the FDA and EMEA guidance differ by a factor of 10 when dilution is taken into account. “The way the two guidelines express this trigger may be confusing,” says Virginia Cunningham, director of environmental sustainability sciences for GlaxoSmithKline. She explains that the EMEA’s trigger of 0.01 microgram per liter (μg/L) reflects a surface water concentration, whereas the FDA’s 1.0 μg/L trigger reflects an “expected introduction concentration,” or the concentration of a compound in sewage effluent.

The EMEA trigger of 0.01 μg/L is calculated from the maximum daily dose of the drug per patient and the assumption that 1% of the population is treated daily with the drug; this is divided by the amount of waste-water per person per day and a dilution factor of 10. The FDA trigger corresponds to a PEC in surface water of 0.1 μg/L, assuming a dilution factor of 10, and is calculated from manufacturers’ sales estimates.

The consideration given to metabolites and the provision for the introduction of scientific experts into the risk assessment process—both part of the revisions to the 2003 guidance—are welcomed by scientists. “It allows for experts to be drawn into the discussion and give their opinions rather than be sticking blindfolded to a number,” says Evelyn O’Brien, a scientist in the Ecotoxicology Workgroup at the University of Konstanz in Germany and coauthor of a discussion of the draft guideline published in the July 2004 *Trends in Biotechnology*.

One caution added by zoologist Theo Colborn, whose seminal 1996 work *Our Stolen Future* uncovered the dangers of endocrine disruptors in the environment, is that conflict of interests for experts working in academia but funded by drug companies must be revealed. “The important thing is,” she says, “that in [the United States] they’re selecting experts to do things like this on campuses where the particular department that that individual is working in oftentimes receives tremendous amounts of grant money from the pharmaceutical company. Openly admitting conflict of interest is so important.”

The EMEA website notes that members of the agency’s scientific committees “are not permitted to have any direct financial or other interests in the pharmaceutical industry. . . . They are required to make an annual declaration of their financial interests and also any indirect interests which could relate to the pharmaceutical industry.” Colborn also hails the guidance for including excipients as well as active ingredients in the risk assessment process. For instance, phthalates such as diethyl phthalate and dibutyl phthalate, used as plasticizers in the coating of some site-directed drugs, may be a potential source of phthalates for people taking these drugs, as reported in the May 2004 issue of *EHP*.

## Limitations of the Guidance

There are certain serious, though perhaps unavoidable, limitations to the guidance. One is the fact that they are not retroactive. “The only thing that [researchers] are concerned about is that the guidance only concerns those pharmaceuticals that are not yet on the market,” says Heberer. “It’s our main concern about this guideline, but compared to the situation in the past it’s really an advance.” But even if future legislation required the environmental risk assessment of drugs already on the market, the big question would be who should do the testing since the originator of a drug is often no longer the main manufacturer.

Another major problem is that monitoring may be difficult. “There are problems detecting certain substances that have been on the market for years,” says O’Brien. Examples of such hard-to-detect drugs include the antidepressants known as selective serotonin reuptake inhibitors (which include Paxil, Prozac, and Zoloft). “So the analysis can be quite difficult,” she says, “and that’s one of the main stumbling features.”

Further, it is not clear how drugs that pose risks will be handled, apart from the addition of labels to recommend appropriate disposal of expired drugs. Another emerging area of concern in North America and Europe alike is the disposal of used birth control patches and hormone replacement patches. Because pharmaceuticals can save lives, the guidance does not suggest removing them from the market even when a risk is found.

“I think there’s going to be a lot of emphasis on labeling, and also on treatment processes,” says Alistair Boxall, a senior lecturer at York University and Central Science Laboratory in England. “So perhaps if you’ve got a hospital where cancer drugs are being used, it may be that we have to start putting treatment processes on the end of the [sewer] pipes of those hospitals to remove some of the drugs.”

Drug take-back programs for expired pharmaceuticals are in place in parts of Europe, so labeling drugs with instructions to return unused portions to a pharmacy makes sense. By comparison, in the United States, the Controlled Substances Act complicates such schemes because it prohibits patients from transferring controlled medicines to anyone other than a law enforcement official. However, a drug return program has recently been legislated (though not implemented) in Maine.

Another limitation, also difficult to avoid, is that the draft guidance only briefly addresses the possibility of additive or synergistic effects, noting that an assessment factor of 10 is applied to the PNEC to account for extrapolation from lab data to field impacts. “It’s worth pointing out that the guidance is written as if the concern is for a single drug in isolation,” says Christian Daughton, chief of the environmental chemistry branch at the Environmental Protection Agency National Exposure Research Laboratory. “But if a drug shares a common mechanism of action with other drugs, or even other pollutants, there’s the possibility for additive effects.”

Some scientists and drug companies are concerned that assumptions in the guidance could lead to unrealistic PECs. The initial calculation assumes the worst-case scenario: that the drug is not metabolized or degraded at all, so the full dose ends up in the environment (this is one of 30 points raised by the Pharmaceutical Research and Manufacturers of America in their comments on the guidance). But others worry that actual concentrations in the environment could be higher than the calculated PEC due to the guidance’s assumed 1:10 dilution factor for sewage effluent entering rivers. In farming areas, water levels drop precipitously in dry weather when water is drawn for crops and cattle, so the 1:10 dilution factor could be too high. Colborn, a Colorado resident, says, “Most of the river water that’s in this part of the West is coming from returned sewage treatment plants.” O’Brien argues the same point in cities where the influx of people stretches the capacity of sewage treatment plants.

Another problem noted by O’Brien is that peak or seasonal variations are not taken into account—flu epidemics, drought, or heavy snowfall could temporarily increase drug concentrations in specific places to values higher than the calculated PEC. Colborn also comments that local use of pharmaceuticals differs, reflecting, for example, recent visits by pharmaceutical representatives telling doctors about new drugs. “To estimate that pharmaceuticals will be released homogeneously across a particular region is, I think, mistaken,” she says. Daughton addressed these and related issues in greater detail in the May 2003 issue of *EHP*.

One worry for pharmaceutical companies is that the increased amount of testing required could translate into costly delays for the release of new drugs. About 50 new drugs come onto the market in the United States each year, and approximately a dozen of those are predicted to occur above the trigger concentration requiring them to undergo the first level, or Tier A, of risk assessment testing.

But only one new drug in the last few years has gone on to the next level to be tested for environmental risks through chronic ecotoxicity tests, according to Florian Zielinski, a chemist at the FDA Center for Drug Evaluation and Research. “In fact, in the States, almost all pharmaceuticals in the Tier A assessment will come out at under one microgram per liter,” says Chris Metcalfe, a professor in the Environmental and Resource Studies Program at Trent University in Ontario, “whereas in the EU there will be a fair number of pharmaceuticals which will move from the Tier A to the Tier B as a result of their lower thresholds.” British labs put about 20 new pharmaceutical products on the market each year.

## Forging Ahead

Since neither the EMEA guidance nor its U.S. sister document addresses pharmaceuticals already on the market, there is much research into whether wastewater treatment can economically remove pharmaceuticals. Increased retention time within treatment plants, chlorination, ozonation, and the natural reduction of a compound’s mass or concentration over time due to processes such as biodegradation all increase the removal of some drugs from wastewater; more advanced treatments such as adding activated carbon or reverse osmosis can remove even more. “But there’s never a silver bullet,” says Shane Snyder, research and development project manager of the Southern Nevada Water Authority. “There’s always a catch.”

The catch with ozone treatment is that it forms bromate, which is a regulated disinfection by-product; with chlorination, the catch is that chlorine combines with ammonia in the sewage treatment system to form chloramines, which are not strong oxidants and so cannot break down compounds such as estrogens. However, chlorination can destroy almost all the estrogens if ammonia is removed first, says Snyder. But even with the use of reverse osmosis (which removes pharmaceuticals down to parts per trillion) and the addition of activated carbon, there’s the problem of what to do with the retained contaminants.

Although Europe has been at the forefront of recognizing and addressing the potential environmental hazard posed by pharmaceuticals, other countries are perhaps beginning to catch up. In the United States, for example, the Federal Interagency Task Group on Pharmaceuticals and Personal Care Products was formed in September 2004. This group comprises seven federal agencies and is chaired by the FDA. The group had its first face-to-face meeting in July 2005 to identify federal research needs and gaps. One of the questions raised was how much of the estrogen in wastewater comes from synthetic sources.

In Canada, the Environmental Impact Initiative was formed in 2001 in response to growing evidence that pharmaceutical substances are being found in the environment. The initiative, which accepted public comments through September 2005 on proposed options for regulating these substances, may result in new rules for the environmental assessment of substances in products regulated under the Food and Drugs Act, according to Health Canada. Japan is also in the process of formulating a plan for environmental risk assessment of pharmaceuticals with sales exceeding one ton per year.

In the meantime, the EMEA draft guidance is seen as an appropriate response to an emerging issue which includes possible risks not just from pharmaceuticals but also from personal care products. “What has come into the scientific literature is that most pharmaceuticals do not show acute ecotoxicity, so the whole mindset is shifting to chronic toxicity, and I think the EMEA guideline is trying to reflect that,” says Cunningham. “None of the people I talk to have a problem with that.”

## Figures and Tables

**Figure:**